# Rice Chloroplast Genome Variation Architecture and Phylogenetic Dissection in Diverse *Oryza* Species Assessed by Whole-Genome Resequencing

**DOI:** 10.1186/s12284-016-0129-y

**Published:** 2016-10-18

**Authors:** Wei Tong, Tae-Sung Kim, Yong-Jin Park

**Affiliations:** 1 Department of Plant Resources, College of Industrial Sciences, Kongju National University, Yesan, 32439 Republic of Korea; 2 Department of Agricultural Sciences, College of Natural Sciences, Korea National Open University, ﻿Seoul, 0308﻿7 Republic of Korea; 3 Center for Crop Genetic Resource and Breeding (CCGRB), Kongju National University, Cheonan, 31080 Republic of Korea

**Keywords:** Chloroplast, African rice, Phylogenetic, Asian rice, Resequencing, Variation

## Abstract

**Background:**

Chloroplast genome variations have been detected, despite its overall conserved structure, which has been valuable for plant population genetics and evolutionary studies. Here, we described chloroplast variation architecture of 383 rice accessions from diverse regions and different ecotypes, in order to mine the rice chloroplast genome variation architecture and phylogenetic.

**Results:**

A total of 3677 variations across the chloroplast genome were identified with an average density of 27.33 per kb, in which wild rice showing a higher variation density than cultivated groups. Chloroplast genome nucleotide diversity investigation indicated a high degree of diversity in wild rice than in cultivated rice. Genetic distance estimation revealed that African rice showed a low level of breeding and connectivity with the Asian rice, suggesting the big distinction of them. Population structure and principal component analysis revealed the existence of clear clustering of African and Asian rice, as well as the *indica* and *japonica* in Asian cultivated rice. Phylogenetic analysis based on maximum likelihood and Bayesian inference methods and the population splits test suggested and supported the independent origins of *indica* and *japonica* within Asian cultivated rice. In addition, the African cultivated rice was thought to be domesticated differently from Asian cultivated rice.

**Conclusions:**

The chloroplast genome variation architecture in Asian and African rice are different, as well as within Asian or African rice. Wild rice and cultivated rice also have distinct nucleotide diversity or genetic distance. In chloroplast level, the independent origins of *indica* and *japonica* within Asian cultivated rice were suggested and the African cultivated rice was thought to be domesticated differently from Asian cultivated rice. These results will provide more candidate evidence for the further rice chloroplast genomic and evolution studies.

**Electronic supplementary material:**

The online version of this article (doi:10.1186/s12284-016-0129-y) contains supplementary material, which is available to authorized users.

## Background

The chloroplast is maternally inherited in most angiosperms and possesses its own genome encoding many chloroplast-specific components (Hagemann 2010; Palmer et al. 1988; Sugiura 1989). The chloroplast has a circular genome, ranging in size from 39.4 to 200.8 kb among photosynthetic plant species (Kohler et al. 1997; Turmel et al. 1999). More than 800 eukaryotic *viridiplantae* chloroplast genomes have been described to date (http://www.ncbi.nlm.nih.gov/genomes/GenomesGroup.cgi?taxid=2759&opt=plastid). The chloroplast genome sequence of rice Nipponbare (*O. sativa* L. ssp. *japonica*) was reported to have a length of 134,525 bp (Hiratsuka et al. 1989). Chloroplasts contain both highly conserved genes fundamental to plant life and more variable regions, which have been informative over broad time scales. Comparative studies of the genomic architecture showed that the order of genes and the contents of essential genes are highly conserved among most chloroplast genomes (De Las Rivas et al. 2002; Kato et al. 2000). Nevertheless, variations among different and closely related genomes have occurred during evolution (Provan et al. 1997; Tang et al. 2004).

The availability of rice nuclear (Goff et al. 2002; Yu et al. 2002) and chloroplast (Hiratsuka et al. 1989) reference genomes has enabled detailed studies of the origin, domestication, and phylogenetic relationships within this group. In particular, whole chloroplast genome analysis provides high-resolution plant phylogenies (Parks et al. 2009). Due to the high level of conservation, analysis of the chloroplast genome has become a valuable tool for plant phylogenetic studies (Waters et al. 2012; Yang et al. 2013). Previously, only a few chloroplast markers have been applied in studies of plant diversity and evolution (Ishii et al. 2001; King and Ferris 2000; Schroeder et al. 2011; Soejima and Wen 2006). From the conventional sequencing of plant chloroplast genomes to next-generation sequencing (NGS), it has become increasingly feasible to investigate the entire genome of the chloroplast, rather than targeting individual regions (McPherson et al. 2013; Nock et al. 2011; Straub et al. 2012). Whole chloroplast genome sequencing for phylogenetic analysis without prior isolation or amplification is now relatively straightforward for plant species (Nock et al. 2011). However, the chloroplast genome only represents the maternal evolutionary history. In addition, it also cannot be fully applied to rapidly diverging taxa, as the chloroplast has a slow rate of evolution (Moore et al. 2010; Parks et al. 2009). Therefore, chloroplast-based evolutionary studies must sometimes be complemented by nuclear genomic information.

Asian cultivated rice (*O. sativa* L.) is generally considered to have been domesticated from *Oryza rufipogon* several thousands of years ago (Cheng et al. 2003; Huang et al. 2012; Khush 1997; Oka 1988). However, there has been some debate regarding the origin of cultivated rice over the past several years, which centered on whether the two major rice cultivars, *O. sativa* L. ssp. *indica* and *japonica*, were derived from a single ancestor or were domesticated independently at different locations (Jin et al. 2008; Kawakami et al. 2007; Li et al. 2006; Molina et al. 2011; Zhang et al. 2009; Zhu et al. 2011; Huang et al. 2012; Xu et al. 2012; Zhu and Ge 2005). While, African cultivated rice (*O. glaberrima*), which was thought to be domesticated from the wild progenitor *O. barthii* ~3000 years ago, had been demonstrated to be domesticated in a single region along the Niger river with independent and distinct process in regard to Asian cultivated rice (Wang et al. 2014). A closer evolutionary relationship between *indica* and *aus* strains were observed using both nuclear and chloroplast genome data, as well as among the *tropical japonica*, *temperate japonica*, and *aromatic* groups (Garris et al. 2005). The *indica* subpopulation was shown to contain the highest degree of chloroplast diversity (Garris et al. 2005). Kim et al. (2014) evaluated 67 improved varieties and 13 landraces from the Democratic People’s Republic of Korea (DPRK) at both nuclear and chloroplast levels, and they found a *temperate japonica* subgroup that was less diverse than the *indica* ancestor group at the nuclear level but more diverse at the chloroplast level (Kim et al. 2014). Whole chloroplast genome phylogenetic analysis revealed that the *Oryza nivara* is closed to *O. sativa* L. spp. *indica* and the *O. sativa* L. spp. *japonica* is closed to *Oryza rufipogon* in Asian cultivated and wild rice (Brozynska et al. 2014) and the African rice (*Oryza glaberrima* and *Oryza barthii*) were cluster together but in separate group with the Asian rice (Wambugu et al. 2015). Our previous studies indicated that the use of chloroplast genome variation to study diversity, population genetics, and phylogenetic analysis was quite convincing and also supported some previous outcomes (Tong et al. 2015). Despite these chloroplast-related studies, a large number of accessions must be applied to extend these studies from limited collections and specific varieties. In addition to rice, chloroplast genome-dependent phylogenetic analyses have also been performed in apple, tangerine, and other species. (Nikiforova et al. 2013; Carbonell-Caballero et al. 2015).

In the present study, a collection of 383 rice accessions with diverse ecotypes, including Asian cultivated and wild rice (*O. sativa* L. spp. *indica* and *japonica*, *Oryza rufipogon*, *Oryza nivara*) and African cultivated and wild rice (*Oryza glaberrima*, *Oryza barthii*) were selected to investigate the variation, diversity, and phylogenetic of rice chloroplast genome. The chloroplast genome of *O. rufipogo*n [Genbank: NC_017835], which is thought to be the immediate ancestral progenitor of cultivated rice, was chosen as the reference. Chloroplast variations in the collection were mined and subjected to comparative analysis among different groups. Diversity, population structure, and principal component analysis were also performed in the current collection. Phylogenetic analysis that conducted using the maximum likelihood (ML) and Bayesian inference (BI) methods and population splits evaluation were investigated, which could provide evidence to illustrate the phylogenetic relationships among rice subgroups, with a focus on Asian cultivated rice, as well as African rice (*Oryza glaberrima* and *Oryza barthii*). This report provides a further case study for the rice chloroplast genome, and the data generated here could be applied to further analyses of rice chloroplast evolution and genetics.

## Results

### Re-Sequencing and Variation Architecture Across the Chloroplast Genome

In this study, we re-sequenced 295 accessions of Asian cultivated rice with a high mean coverage (~7.34×), generating ~920Gbp raw sequence base with ~9.18 billion reads. After removing the low quality bases, a total of ~8.89 billion clean reads (with a clean read rate of 96.96 %) and ~860Gbp clean bases (with a clean base rate of 93.73 %) were obtained (Additional file 1: Table S1). Then this data was carried out for rice chloroplast genome variations detecting and phylogenetic analysis together with other 88 rice accessions.

**Fig. 1:**
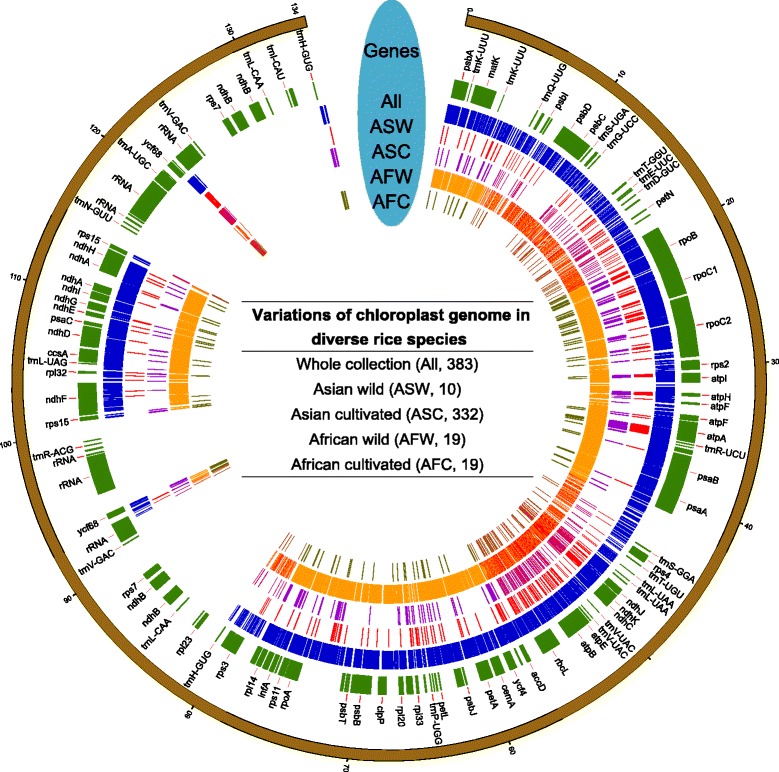
Overall distribution of variations (SNPs and indels) across the chloroplast genome. Variations of all 383 accessions and different groups were identified based on the chloroplast reference genome of *Oryza rufipogon*. Circular map showing the chloroplast genome structure. The unit of the outer distance is kb. “Genes” indicating the genes (including genes, tRNA and rRNA) with their locations on the reference (*O. rufipogon*) genome. Each colored line indicating one variation. ASW: Asian wild rice, ASC: Asian cultivated rice, AFW: African wild rice, AFC: African cultivated rice. The numbers in the brackets of middle table indicated the number of accessions

Variations in 383 rice accessions, including 335 Asian cultivated rice (*O. sativa* L.), 10 Asian wild rice (*O. rufipogon, O. nivara*), 19 African cultivated rice (*Oryza glaberrima*), and 19 African wild rice (*Oryza barthii*), were characterized based on whole-genome resequencing data using the chloroplast genome of *O. rufipogon* as a reference. A total of 3677 variations, including 3592 SNPs and 85 indels (insertions/deletions), were identified in the whole collection (Table 1). A variation density of 27.33 per 1kb were observed through the total SNPs/indels. However, after excluding missing genotypes with MAF (Minor Allele Frequency) ≥ 0.01, high-quality (HQ) variations were dramatically decreased to 242, including 227 SNPs (93.8 % of the total HQ variations) and 15 indels (6.2 % of the total HQ variations) with a variation density of 1.8 per 1 kb (Table 1). The overall variations across the genome and groups specific variations were also extracted, suggesting that the African wild rice hold about 82.9 % of the total variations on its own (Fig. 1, Table 1). What’s more, the distribution of the variations across the chloroplast genome is uneven (Fig. 1). Except the African wild rice, which harbored 2982 HQ variations (97.8 % of all variations), the Asian wild rice possessed the most number of HQ variations, even with only 10 accessions. Interestingly, a greatest abundance of variations in the African wild rice were observed both in all and HQ variations among all of the groups, however, the African cultivated rice had minimal variations.

**Table 1:** Summary of the total variations (SNPs/indels) detected in the germplasm and subgroups and the location distribution of the variations

Group	All variations				HQ variations^a^			
	Total	SNPs	Indels	Density/kb	Total	SNPs	Indels	Density/kb
Whole collection (383)^b^	3677	3592	85	27.33	242	227	15	1.8
Asian cultivated (332)^c^	723	671	52	5.37	308	281	27	2.29
Asian wild (10)	413	374	39	3.07	354	321	33	2.63
African cultivated (19)	418	385	33	3.11	280	255	25	2.08
African wild (19)	3049	3016	33	22.66	2982	2958	24	22.16
	Genic^d^	Intergenic		Genes^e^	Genic	Intergenic		Genes
Whole collection (383)	2156	1521		87	141	101		27
Asian cultivated (332)	312	411		45	149	159		28
Asian wild (10)	187	226		37	167	187		32
African cultivated (19)	171	247		35	108	172		29
African wild (19)	1948	1101		86	1915	1067		81

^a^HQ variation: High-quality variation, referring to variations excluding missing genotypes and MAF ≥ 0.01

^b^Subgroups in the whole collection. The numbers in brackets indicate the number of accessions

^c^Three mixed accessions belong to Group III, IV and V in the 50 cultivated and wild rice group were excluded here

^d^The genic region also includes tRNAs and rRNAs. ^c^The total number of genes that the variations harbored

After determination of the location of variations across the genome, 2156 SNPs/indels were found within the genic region scattered over 87 genes in whole variations, including those encoding tRNAs and rRNAs (Table 1, Additional file 2: Table S2). Only 141 variations were retained by HQ selection in the genic region, involved 27 genes. In the four different groups, maximum variations in African wild rice were found both in all and HQ variations, as expected, which including 86 and 81 genes, respectively. In HQ variations, the Asian wild rice held the most number of genic variations and involved genes except the African wild rice.

Different allele types were also investigated, which indicated that T/C and A/T have the most number in all variations, while A/G and C/T are the major types in HQ variations. The overall Ts/Tv (Transition/Transversion) ratio in chloroplast genome of whole collection was 0.7328, which indicates that the mutations within the same type of nucleotide were less than those from a pyrimidine to a purine or vice versa (Additional file 3: Figure S1). In the four groups, the Asian wild rice holds the highest Ts/Tv ratio (1.047), while the African wild rice holds the lowest (0.7093).

### Genetic Diversity Evaluation of Rice Chloroplast Genome

**Fig. 2:**
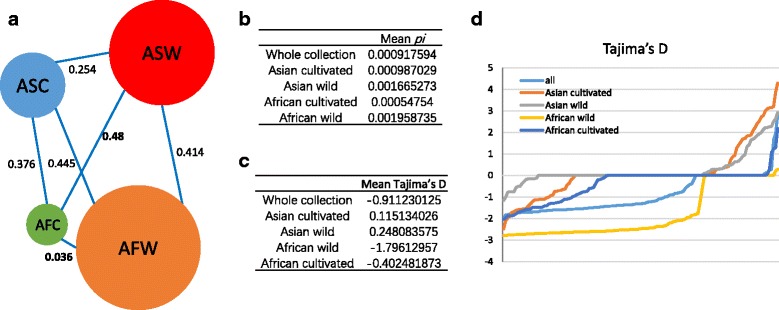
Chloroplast genome nucleotide diversity, genetic distance (*Fst*), and Tajima’s *D* test. **a** Nucleotide diversity (*pi*) and *Fst* value of four groups. The four circle indicated four groups and the circle size indicated the *pi* value. The *Fst* value between each two groups were represented by the distance between them. **b**, **c** Average *pi* and *Tajima’s D* value of the whole collection and each group. **d** Tajima’s *D* test of the overall chloroplast genome in 1kb bins. The sorted values were plotted in each groups. ASW: Asian wild rice, ASC: Asian cultivated rice, AFW: African wild rice, AFC: African cultivated rice

The nucleotide diversity (*pi*) of the whole collection and different groups (Asian cultivated and wild rice, African cultivated and wild rice) was calculated with a mean *pi* of 0.000918 in whole collection. While among the subgroups, the African wild rice has the highest diversity (0.001959), and the African cultivated rice has the lowest (0.000548) (Fig. 2a, b, Additional file 4: Table S3). The Asian wild rice also holds a high *pi* (0.001665), and the Asian cultivated rice has the similar *pi* with whole collection (0.000987).

A long genetic distance (*Fst*) between Asian rice and African rice was observed (~0.43), which indicates the low levels of breeding and low connectivity between them (Fig. 2a). The African cultivated rice (*O. glaberrima*) has very high breeding level with the African wild rice (*Oryza barthii*). These results may suggest and support the distinct domestication between African and Asian rice. Tajima’s *D* value of the chloroplast genome was also examined for detection of balancing selection (Fig. 2c, Additional file 5: Table S4). The negative value indicated population size expansion and/or purifying selection, while a positive value indicated a decrease in population size and/or balancing selection. Values closer to 0 indicate less evidence for the occurrence of selection. According to the distribution (Fig. 2d), Tajima’s *D* value of all groups showed a location fluctuation in 1kb bins with positive, negative value and also 0. Excepting the African wild rice and whole collection, which showing more negative positions, other groups showing a relative even distribution of positive, negative and 0. The mean Tajima’s D value of whole collection and different groups was shown in Fig. 2c, the whole collection (because of diverse rice accessions) and African wild rice showing a relative high divergence. While closer Tajima’s *D* value to 0 indicated rare selection in the chloroplast genome.

### Population Structure and Principal Component Analysis Based on Chloroplast Genome

**Fig. 3:**
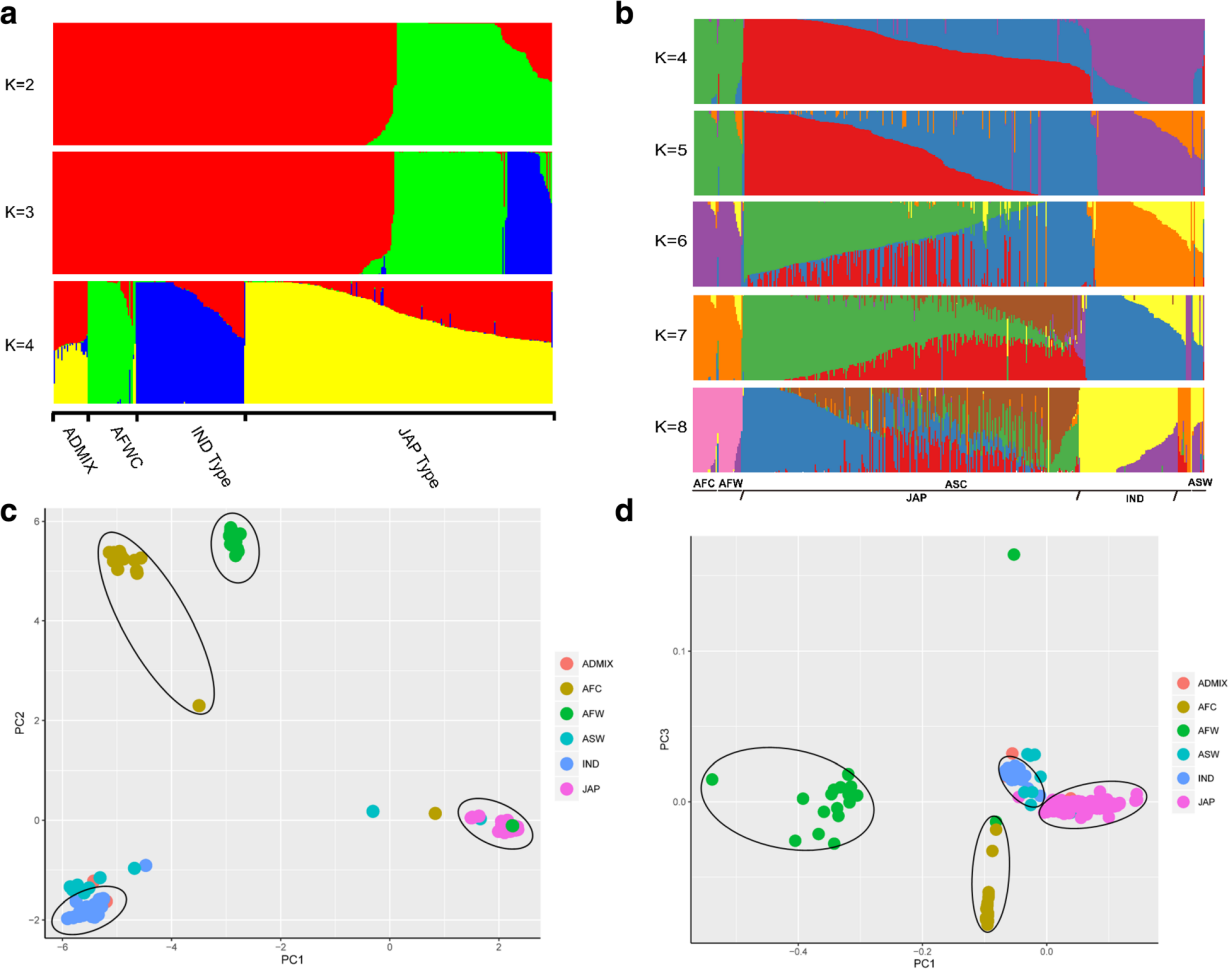
Population structure and principal component analysis of the collection. **a** Population structure clustering using the high-quality variations with an optimal *K* value of 4. **b** Validation of structure clustering using ADMIXTURE software. Here, an optimal *K*  =  8 was defined. Only the structure from 4 to 8 were displayed here, since the results from K =2 to 3 were same with the results from STRUCTURE. **c** Principal component analysis of all of the accessions. **d** Multidimensional scaling (MDS) analysis of the whole collection, which can be used to prove the result from PCA. ASW: Asian wild rice, ASC: Asian cultivated rice, AFW: African wild rice, AFC: African cultivated rice. ADMIX in (**a**) represents the not clearly separated accessions. In (**c**) and (**d**), ADMIX indicated the three mixed accessions in the 50 cultivated and wild rice (Xu et al. 2012)

The population structure of the whole collection was investigated based on the HQ variations using SRUCTURE, which estimates individual ancestry and admixture proportions assuming *K* populations. With increasing *K* (number of populations) values from 1 to 10 with 10 iterations each, we analyzed the population structure for each *K* value (Fig. 3a, from *K* = 2 to 4). We distinguished the major substructure groups using an optimal *K* value of 4 (highest *ΔK*, Additional file 6: Figure S2a). All the collected accessions formed four subpopulations, denoted as *indica* type, *japonica* type, Admixture, and African rice (wild and cultivated). In addition, a validation of population structure was conducted using ADMIXTURE from *K* = 1 to 10. With a cross-validation procedure, a good *K* value of 8 was adopted, which exhibited a lowest cross-validation error in all *K* values (Additional file 6: Figure S2b). The population structure form *K* = 4 to 8 was illustrated (Results in *K* = 2 and 3 were almost same using SREUCTURE and ADMIXTURE), which clustered the population into four subgroups (African cultivated and wild, Asian wild, and Asian cultivated with *indica* and *japonica* type) tightly (Fig. 3b, from *K* = 4 to 8). The results were consistent using two software, which indicates the clear separation of African and Asian rice. A similar clustering within the Asian cultivated rice (*indica* and *japonica*) was also observed, which actually also consistent with the clusters in nuclear genome test.

PCA using the whole variation data was conducted in TASSEL, with the first two PCs explaining more than 81.9 % of the proportional variance; therefore, we constructed the PCA using PC1 and PC2 (Fig. 3c). Four main groups were inferred, *indica* type, *japonica* type, African wild and cultivated rice, as well as several scattered accessions (Asian wild rice) and admixed among them. Multidimensional scaling (MDS) analysis was also conducted with TASSEL, which reveals four major groups that were almost same with PCA result (Fig. 3d). Even though no perfect clustering was found according to nuclear genome structure, these variations and the present case study also suggested that chloroplast genome-based analyses can be applied in population genetics studies.

### Rice Phylogeny Based on the Chloroplast Genome

**Fig. 4:**
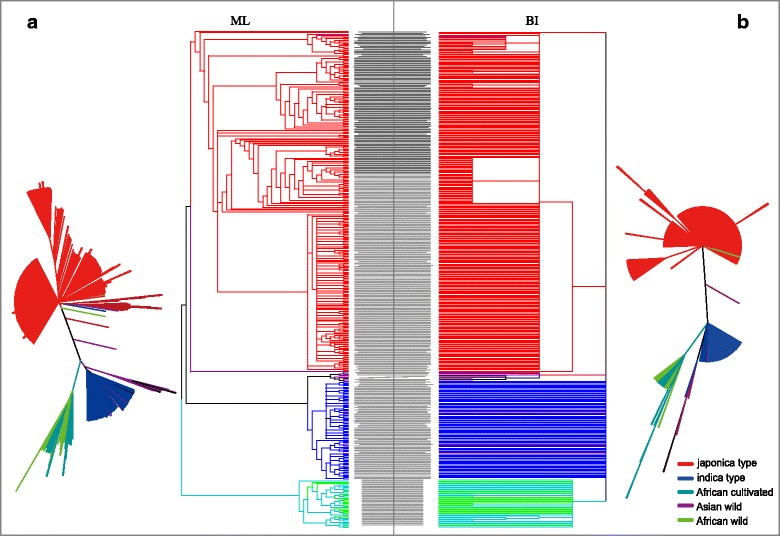
A tanglegram phylogenetic analysis using trees from ML and BI methods to compare the difference of the two methods and illustrate the relationships of the different *Oryza* groups. Here, high-quality variations were applied in both analyses. **a** Phylogram and radial tree layout of the ML tree based on a best-fit model (SYM + G). **b** BI-based tree using the best-fit model JC + G. Best-fit models were evaluated using jModeltest. The tanglegram was implemented in Dendroscope using a Neighbor Net-based heuristic method, which use line connects the same accession in two trees to see the difference phylogenetic structure

Phylogenetic analysis of the whole rice collection was performed using a ML iterative model-based method with a bootstrap of 1000 replicates to assess the reliability of the phylogeny reconstructed using PhyML. In parallel, phylogenetic analysis was also inferred using a Bayesian MCMC search method. The ML method suggested three clear groups (*indica* type, *japonica* type, and African rice), with the Asian wild rice scattered between *indica* and *japonica* (Fig. 4a). Most of the accessions showed clear separation into the *japonica* group, *indica* type, or African rice group, indicated by the clustering of the 50 cultivated and wild rice accessions. Similar phylogenetic results were also obtained using the BI method displayed in Fig. 4b. A comparison of the trees from two methods was implemented in a tanglegram, which reveals that the overall phylogenetic structure and clustering of the accessions in the two trees are nearly same (the same accession in two trees can connect with each other at the same location in the clusters), even the outward shape of the two trees are not well unified. The i*ndica*, *japonica*, and African rice groups showed almost the same clustering in two methods, but the Asian wild rice showed closer with *indica* group in the BI method. From the results, we inferred that *indica* and *japonica* may have an independent domestication, as the Asian wild rice was clustered between them (4 of the wild rice are mixed inside the *indica* and *japonica* group). Meanwhile, it was obvious that African rice, including cultivated (*O. glaberrima*) and wild (*O. barthii*) were in an independent group, even the wild and cultivated are not well separated. It also can be inferred that *O. glaberrima* was from *O.barthii* and have an independent domestication process distinct with *O. sativa* L.

**Fig. 5:**
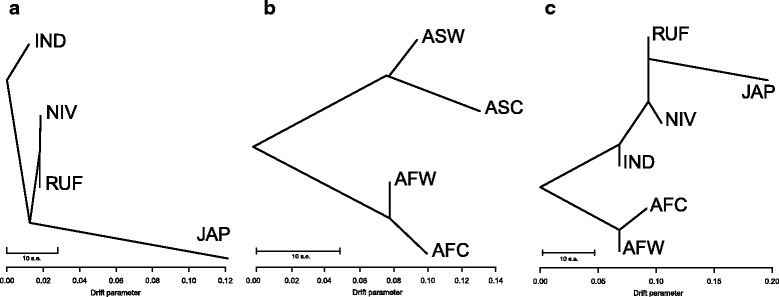
*TreeMix* model inference to evaluate the population splits and mixtures among the populations. **a** Four subpopulations system in Asian rice revealed the relationships of each group. **b** Four subpopulations system in whole collection revealed the relationships between Asian and African rice. **c** Six subpopulations system in whole collection revealed the relationships between Asian and African rice. The scale bar shows ten times the average standard error of the estimated entries in the sample covariance matrix. ASW: Asian wild rice, ASC: Asian cultivated rice, AFW: African wild rice, AFC: African cultivated rice, RUF: Asian wild rice *O.rufipogon*, NIV: Asian wild rice *O.nivara*, IND: *indica* type in Asian cultivated rice, JAP: *japonica* type in Asian cultivated rice

In the *TreeMix* test, the subpopulation relationships were evaluated among two subsets with four and six subpopulations, which revealed that the Asian cultivated rice (*indica* and *jaoponica*) may have different origin (Fig. 5a), since the two subgroups located on different side of the Asian wild rice. By evaluating the population splits between Asian and African rice, different domestication process can be inferred since very distinct clustering was observed (Fig. 5b). When six groups were applied, similar results were obtained, and in addition, the *indica* is closer to the *O. nivara* and the *japonica* is closer to *O.rufipogon* (Fig. 5c).

Together with the results of previous studies regarding the origins of rice, we concluded that *O. sativa* L. spp. *indica* may have evolved from *O. nivara*, and that *O. sativa* L. spp. *japonica* may be domesticated from *O. rufipogon*. Simultaneously, African cultivated rice may have a different and separated domestication process with Asian cultivated rice.

## Discussion

### Genetic Variation and Population Structure in Chloroplast Genome Level

**Fig. 6:**
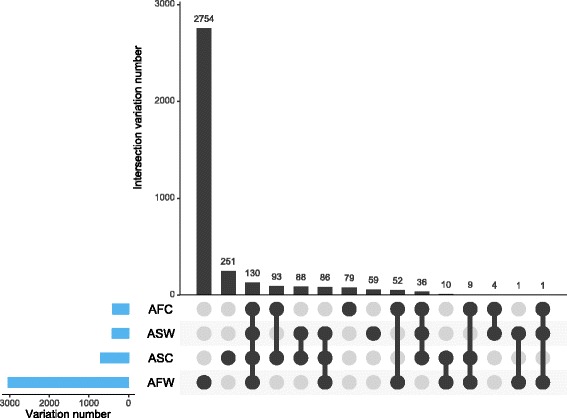
Matrix layout for all intersections of four groups (ASW, ASC, AFW, AFC). Dark histogram indicated the specific variations in one group or intersected variations among two or more groups. Dark circles in the matrix indicate sets that are part of the intersection. The left set indicated the number of variations in each group. ASW: Asian wild rice, ASC: Asian cultivated rice, AFW: African wild rice, AFC: African cultivated rice

Chloroplast DNA shows a much lower substitution rate than does nuclear DNA, which is significantly reduced even in the inverted repeat regions (Wolfe et al. 1987). The overall sequence differences among rice subspecies varieties is ~130-fold higher in the nuclear than chloroplast genomes (0.12 %) (Yu et al. 2002). Therefore, in practice, detecting useful polymorphisms at the population level is difficult, due to the low substitution rates in plant chloroplast genomes. Highly accurate whole-genome sequencing and reference genome based assembly of chloroplast genome become a more economical approach and can be used for the further genomic studies (Wu et al. 2012). In this case, investigating the variations of chloroplast genome based on higher genome coverage sequencing could decrease the number of missing values and heterozygotes, and thus obtain more accurate results. In this report, we evaluated the chloroplast genome variations in a diverse collection of 383 rice accessions with relative high coverage re-sequencing, as well as the variation distribution in different groups (Table 1, Fig. 1). Intersection of variations in different groups was characterized, and only 130 variations were overlapped in four groups. While the African wild rice shown much more total and unique variations than other groups, which may indicate the huge difference between African wild and Asian rice (Fig. 6). And very few overlaps were found that only in African cultivated &Asian wild &African wild, Asian wild &African wild, African cultivated &Asian wild. Besides, considering the HQ variations, the Asian wild rice has the most variations except the African wild rice, inferring that wild type has much higher diversity than cultivated type (Table 1). Moreover, the variations showed a heterogeneity across the chloroplast genome, which leads to no variations in some specific regions (Fig. 1). The average *pi* of the overall genome was low (~0.0009), as were those in other groups, while the wild rice showed higher diversity than their cultivated type. A high *Fst* value (>0.37) was observed between the Asian and African rice, indicating their far genetics distance. Tajima’s *D* test in chloroplast level of African rice showed a negative value, which may indicate some purifying selection or a signature of a recent population expansion. Whereas, the Asian rice that have a positive value may indicate an over-dominant selection or population bottleneck.

The results of the population structure analysis indicated that population clustering based on chloroplast genomes was consistent with the results based on nuclear genomes in most accessions. Besides 2 admixed accessions from the 50 cultivated and wild rice group (Group III and IV), we also found 1 *indica* accession, 2 African accessions, are clustered into or close to *japonica* and several Asian wild accessions scattered between *indica* and *japonica* were observed to be closer to *indica* (Fig. 3c, d). Fortunately, African rice and Asian rice can be well grouped in most accessions at the chloroplast genome level, with well clustering of African wild and cultivated rice. We can infer that the African cultivated rice has distinct genetic background with Asian rice.

### Chloroplast Genome Indicates Independent Origin of *Indica* and *Japonica*

In rice, the evolutionary rate of chloroplast DNA is three-fold higher than that of mitochondrial DNA (Tian et al. 2006). Therefore, its maternal inheritance and relatively high mutation rate are useful for elucidating the phylogeny of the species. The advent of NGS (next-generation sequencing) has allowed detection of substitutions in large populations both easily and accurately, leading to a better understanding in evolutionary studies. It may not be necessary to assemble whole chloroplast genomes for molecular ecology studies by exploring chloroplast variation (McPherson et al. 2013). Chloroplast DNA provides the advantage of a high copy number without recombination, which is a critical issue in nuclear genome-based phylogenetic studies (Poke et al. 2006; Takahashi et al. 2008). Interspecific hybridization can lead to chloroplast capture, whereby the plastome of one species introgresses into another, and this has been used to explain the inconsistencies between chloroplast and nuclear gene trees.

In spite of the debate of the domestication of Asian cultivated rice (*O. sativa* L.), which focus on whether the two major subgroups were in single (Huang et al. 2012; Molina et al. 2011) or independent origins (Londo et al. 2006; Ma and Bennetzen 2004; Vitte et al. 2004; Yang et al. 2012; Zhu and Ge 2005), new opinion about three geographically separate domestications of Asian rice had been proposed recently (Civáň et al. 2015). From these different data and analyzing methods, different or entirely opposite result have obtained. According to Civáň et al. (2015), they got different results using the same data from Huang et al. (2012), which suggested that extreme complicated issues might happen during the long history of rice domestication in nuclear genome level complemented with the response points from Huang and Han (2015) against the new analyzing results. While the chloroplast genome can narrow down this problem for its non-recombination and high level of conservation.

Londo et al. (2006) detected the haplotype network of chloroplast atpB-rbcL region, they concluded that *japonica* rice is less diverse than *indica* rice and also demonstrated that *O. sativa* L. was domesticated from *O. rufipogon* at least twice (Khush 1997; Londo et al. 2006). A recent study of the wild and domesticated rice AA genome species using whole chloroplast genome sequences indicated that the *O. rufipogon* (Asian) and *O. nivara* are always separately clustered with *japonica* and *indica*, respectively (Wambugu et al. 2015). By applied a chloroplast genome-wide variation analysis in current report, we found the evidence support the independent domestication of Asian cultivated rice, *O. sativa* L. spp. *indica* and *japonica*, which were thought to be originated from *O. nivara* and *O. rufipogon*, respectively. According to a chloroplast whole genome sequence investigation from several references, we previously found that the *indica* and *japonica* were closer with *O. nivara* and *O. rufipogon*, respectively, which also indicated the independent origin of Asian cultivated rice (Tong et al. 2015). Population structure of a KRICE_CORE set, which hold 137 accessions in current collection, also supported the hypothesis of the independent origin of *indica* and *japonica* in nuclear genome (Kim et al. 2016). However, as we mentioned previously, the chloroplast genome only represents the maternal evolutionary history, which cannot be fully applied to rapidly diverging taxa. Whereas, in nuclear genome level, different dataset applied with different method sometimes generated different results. Therefore, in some cases, chloroplast genome based evolutionary studies should be complemented with nuclear genome data, and vice versa, to obtain more reliable results.

### Evidence for Distinct Domestication of African Cultivated Rice

*O. glaberrima* was thought to be independently domesticated from the wild progenitor, *Oryza barthii*, ~3000 years ago (Sweeney and McCouch 2007), which is 6000–7000 years after the domestication of Asian rice (*O. sativa* L.) (Vaughan et al. 2008). *O. glaberrima* was domesticated in a single region along the Niger River, as opposed to noncentric domestication events across Africa, which has experienced geographically and culturally distinct domestication processes (Wang et al. 2014). Here, we presented evidence supporting the domestication of *O. glaberrima*, as well as insights into the genetic distance and population structure analyses of the chloroplast genome. In chloroplast *Fst* analyses, the genetic distance value (*Fst*) of African rice, especially African cultivated rice (*O. glaberrima*) with Asian rice was much higher than the distances between the other groups (Fig. 2a), indicating a low level of breeding with the Asian rice. While, a very low value was observed between African cultivated and wild rice. What observed in the population structure and principal component analyses were that African rice always classified as a separated group (Fig. 3c, d), which also can be clearly seen in the phylogenetic trees using both ML and BI methods and in the TreeMix test (Figs. 4 and 5). One more thing we need to note is that in African rice, the cultivated and wild type are not well grouped into only two clusters but intersect, which was also observed in the nuclear genome analysis conducted by Wang et al. (2014). Even though, same conclusion can be inferred according the present result. These observations indicated that *O. glaberrima* was distant from Asian rice and had a distinct domestication process at chloroplast genome level.

## Conclusions

In current report, we described chloroplast variation architecture of 383 rice accessions from diverse regions and different ecotypes. A total of 3677 variations across the chloroplast genome were identified. The chloroplast genome variation architecture in Asian and African rice are different, as well as within Asian or African rice. Wild rice and cultivated rice also have distinct nucleotide diversity or genetic distance. Chloroplast genome nucleotide diversity and genetic distance were investigated, indicated a high degree of diversity in wild rice than in cultivated rice. African rice showed a low level of breeding and connectivity with the Asian rice, suggesting the big distinction of them. Population structure and principal component analysis revealed the existence of clear clustering of African and Asian rice, as well as the *indica* and *japonica* in Asian cultivated rice. Phylogenetic analysis and the population splits test suggested and supported the independent origins of *indica* and *japonica* within Asian cultivated rice. In addition, the African cultivated rice was thought to be domesticated differently from Asian cultivated rice. We hope these results could provide more candidate evidence for the further rice chloroplast genomic and evolution studies.

## Methods

### Samples and Whole-Genome Resequencing

A core set containing 137 rice accessions with diverse types (landrace, weedy, cultivated) previously generated from worldwide varieties collected from the National Genebank of the Rural Development Administration (RDA-Genebank, Republic of Korea) using the program PowerCore (Kim et al. 2007; Zhao et al. 2010; Kim et al. 2016) and 158 bred accessions were selected and sequenced for chloroplast genomic evaluation (Additional file 7: Table S5). In addition, 50 accessions of cultivated and wild rice developed by Xu et al. (Xu et al. 2012) and 19 accessions of African cultivated rice (*O. glaberrima*) and 19 accessions of African wild rice (*O. barthii*) (Wang et al. 2014) were also combined in the present study (Additional file 8: Table S6). Raw data from the 50 cultivated and wild rice, 19 African cultivated rice, and 19 African wild rice accessions were downloaded from the European Nucleotide Archive (http://www.ebi.ac.uk/ena) under accession numbers [SRA023116, SRP038750, and SRP037996] respectively.

For our germplasm (295 accessions with diverse origin), young leaves from a single plant were sampled and stored at –80°C prior to genomic DNA extraction using the DNeasy Plant Mini Kit (Qiagen). Qualified DNA was used for whole-genome resequencing of the collected rice varieties (295 accessions), with an average coverage of approximately 7.34× on the Illumina HiSeq 2000 Sequencing Systems Platform (Illumina Inc.).

### Data preparation, Identification of Variation, and Statistics

Resequencing raw data (Fastq format) of all the accessions were trimmed using Sickle v1.2 (Joshi and Fass 2011) to remove low-quality reads. BWA v0.6.2 (Li and Durbin 2009) was used to align the raw data to the *O. rufipogon* chloroplast genome sequence. A Sequence Alignment/Map (SAM) file was created during the mapping and converted to a binary SAM (BAM) file with sorting. Removal of duplicates and addition of read group IDs were performed using Picard Tools v1.88 (https://broadinstitute.github.io/picard/). Final realignment and identification of variation were performed using GATK software v3 (McKenna et al. 2010). The variant call format file describing the variation result was processed by two python scripts, generating a HapMap (Haplotype Map) file.

Statistical analyses were performed to summarize the number and distribution of single nucleotide polymorphisms (SNPs) and indels (insertions and deletions) based on the HapMap file. The positions of high-quality (HQ, sites without missing and MAF ≥ 0.01, determined by the smallest group number 5 both in *O. nivara* and *O. rufipogon*) SNPs and indels in this population and subgroups were established according to the reference genome of *O. rufipogon*. For the Asian cultivated group, three admixed accessions in the 50 cultivated and wild rice were excluded for the further subgroup comparative analyses.

### Chloroplast Genome Diversity Architecture

Analyses of chloroplast genome nucleotide diversity (*pi*), population divergence (*F*st value), Ts/Tv (Transition/Transversion ratio) and Tajima’s *D* value were conducted using VCFtools (Danecek et al. 2011). Assessments of these calculations in whole collection and different subgroups (Asian cultivated and wild, African cultivated and wild) were performed using VCFtools with a sliding window 1000 bp in length and a 500-bp step size.

### Population Structure and Principal Component Analysis

The population structures of the collection were investigated using the model-based program STRUCTURE v2.3.4 (Pritchard et al. 2000) with a burn-in period length of 100,000 and a Markov chain Monte Carlo (MCMC) rep number of 200,000, which implements a Bayesian approach to identify subpopulations with distinct allelic frequencies and places individuals into *K* clusters. The distribution of L (*K*) revealed a continuously increasing curve without a clear maximum for true *K*. To overcome these difficulties in identifying the true *K* value, an ad hoc quantity (*ΔK*) was calculated based on the second-order rate of change of likelihood (*∆K*) using the software Structure Harvest (Evanno et al. 2005; Earl 2012). Besides, the population structure was also validated using another model-based software ADMIXTURE (Alexander et al. 2009). By using ADMIXTURE’s cross-validation procedure, a good value of K can be obtained, which will exhibit a low cross-validation error compared to other K values. Principal component analysis (PCA) and multidimensional scaling (MDS) was conducted using TASSEL 5 (Bradbury et al. 2007), which could provide more evidence and complement the population structure analyses. MDS produces results that are similar to PCA but starts with a distance matrix and results in coordinate axes that are scaled differently.

### Chloroplast-Based Phylogenetic and Population Splits

ML and BI methods were applied to construct a phylogenetic tree for all 383 accessions. Briefly, appropriate nucleotide substitution models were assessed using jModeltest 2.1.7 (Darriba et al. 2012). A phylogenetic tree was conducted using PhyML 3.0 (Guindon et al. 2010) complemented by the best nucleotide substitution model SYM + G (symmetrical model + gamma distribution) selected by the hierarchical LRT (Hierarchical Likelihood Ratio Test) (Felsenstein 1988) and the Akaike Information Criterion (AIC) (Akaike 1974) with 1000 bootstrap replicates. A Bayesian tree was constructed using MrBayes 3.2.5 (Ronquist et al. 2012) implemented with a Bayesian MCMC search, with two parallel runs of 2 million generations and four chains each. Best-fit model JC + G (Jukes-Cantor + gamma distribution) were selected according to the Bayesian Information Criterion (BIC) (Schwarz 1978). The phylogenetic tree was displayed and modified using Figtree v1.4.2 (http://tree.bio.ed.ac.uk/software/figtree/). The consensus tree of the bootstrap in the ML method was integrated using Phylip software (Phylogeny Inference Package v3.695, http://evolution.genetics.washington.edu/phylip.html). A tanglegram for two trees was implemented in Dendroscope (Huson and Scornavacca 2012) using a Neighbor Net-based heuristic, which is one good way to visualize similarities and differences between two phylogenetic trees side by side connected with lines between taxa that correspond to each other.

Additionally, a *TreeMix* model for inferring the set of population splits and mixtures in the history of a set of populations was performed using genome-wide allele frequency data in TreeMix (Pickrell and Pritchard 2012). In the collection, four (African cultivated and wild, Asian cultivated and wild, as well as the four groups of Asian rice) and six subpopulations (African wild and cultivated rice, Asian wild and cultivated rice, and the *indica* and *japonica* groups in Asian cultivated rice) were implemented to identify the relationships among the populations.

## Additional Files

Additional file 1: Table S1. Summary of the 295 rice whole genome re-sequencing. (XLSX 30 kb)
Additional file 2: Table S2. Location of all the SNPs and Indels and their gene region in the reference detected in this study. (XLSX 121 kb)
Additional file 3: Figure S1. Overall Ts/Tv (Transition/ Transversion ratio) in 1kb bins of the whole collection and different groups. ASW: Asian wild rice, ASC: Asian cultivated rice, AFW: African wild rice, AFC: African cultivated rice. (DOCX 78 kb)
Additional file 4: Table S3. Nucleotide diversity of the overall chloroplast genome with a 1000bp sliding window and 500bp step size. (XLSX 16 kb)
Additional file 5: Table S4. Overall Tajima’s D testing of the chloroplast genome in a 1kb bin. (XLSX 13 kb)
Additional file 6: Figure S2. Magnitude of *ΔK* as a function of *K* and cross-validation error estimation to find the optimal *K* value for the population structure in STRUCTURE and ADMIXTURE. In this case, the maximum value of *ΔK* for all of the accessions was identified as *K*  =  4 in STRUCTURE. While a lowest error value in K = 8 was identified in ADMIXTURE. But the values were similar from K = 5 to 10. (DOCX 58 kb)
Additional file 7: Table S5. The 295 accessions information sequenced by ourselves and subpopulation designations used in this study. (DOCX 31 kb)
Additional file 8: Table S6. Fifty cultivated and wild rice accessions and 38 African rice (including 19 African cultivated rice and 19 African wild rice) accessions used in the chloroplast genome study. (DOCX 21 kb)
