# Supplementary material for: Rice Chloroplast Genome Variation Architecture and Phylogenetic Dissection in Diverse Oryza Species Assessed by Whole-Genome Resequencing
Source: Rice (N Y). 2016 Oct 18;9:57. doi: 10.1186/s12284-016-0129-y (PMC5069220; doi:10.1186/s12284-016-0129-y)
Supplement: Additional file 3: Figure S1. — Overall Ts/Tv (Transition/ Transversion ratio) in 1kb bins of the whole collection and different groups. ASW: Asian wild rice, ASC: Asian cultivated rice, AFW: African wild rice, AFC: African cultivated rice. (DOCX 78 kb) [file 12284_2016_129_MOESM3_ESM.docx]

**
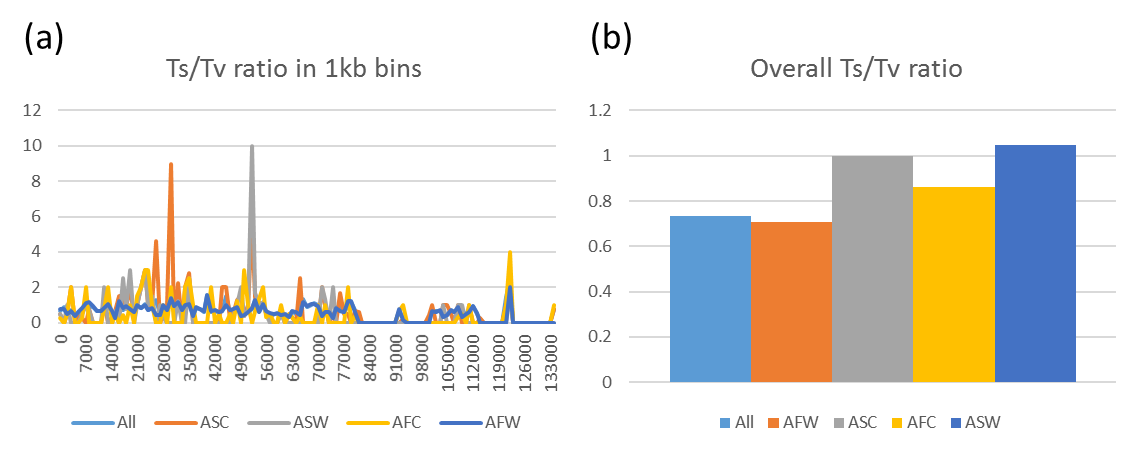
**

**Figure S1.** Overall Ts/Tv (Transition/ Transversion ratio) in 1kb bins of the whole collection and different groups. ASW: Asian wild rice, ASC: Asian cultivated rice, AFW: African wild rice, AFC: African cultivated rice.
