# Supplementary material for: Rice Chloroplast Genome Variation Architecture and Phylogenetic Dissection in Diverse Oryza Species Assessed by Whole-Genome Resequencing
Source: Rice (N Y). 2016 Oct 18;9:57. doi: 10.1186/s12284-016-0129-y (PMC5069220; doi:10.1186/s12284-016-0129-y)
Supplement: Additional file 6: Figure S2. — Magnitude of ΔK as a function of K and cross-validation error estimation to find the optimal K value for the population structure in STRUCTURE and ADMIXTURE. In this case, the maximum value of ΔK for all of the accessions was identified as K = 4 in STRUCTURE. While a lowest error value in K = 8 was identified in ADMIXTURE. But the values were similar from K = 5 to 10. (DOCX 58 kb) [file 12284_2016_129_MOESM6_ESM.docx]

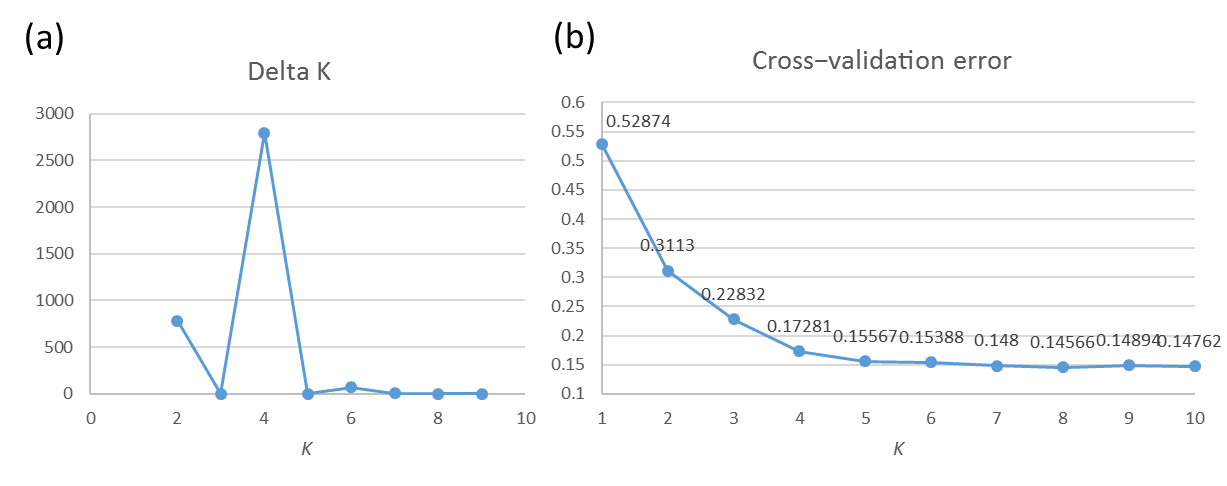


**Figure S2.** Magnitude of *ΔK* as a function of *K* and cross-validation error estimation to find the optimal *K* value for the population structure in STRUCTURE and ADMIXTURE. In this case, the maximum value of *ΔK* for all of the accessions was identified as *K* = 4 in STRUCTURE. While a lowest error value in K = 8 was identified in ADMIXTURE. But the values were similar from K = 5 to 10.
