# Supplementary material for: Rice Chloroplast Genome Variation Architecture and Phylogenetic Dissection in Diverse Oryza Species Assessed by Whole-Genome Resequencing
Source: Rice (N Y). 2016 Oct 18;9:57. doi: 10.1186/s12284-016-0129-y (PMC5069220; doi:10.1186/s12284-016-0129-y)
Supplement: Additional file 7: Table S5. — The 295 accessions information sequenced by ourselves and subpopulation designations used in this study. (DOCX 31 kb) [file 12284_2016_129_MOESM7_ESM.docx]

**Table S5.** Information of the KAS accessions and its subpopulation designation used in this study.

| **No** | **Accession name** | **Origin** | **Type ^a^** | **Subspecies ^b^** |
| --- | --- | --- | --- | --- |
| Acc-001 | NAMSEON 52 | Korea | Breeding line | *japonica* |
| Acc-002 | SUWEON 159 | Korea | Breeding line | *japonica* |
| Acc-003 | SUWEON 255 | Korea | Breeding line | *Indica* |
| Acc-004 | SUWON 301 | Korea | Breeding line | *japonica* |
| Acc-005 | Suweon 347 | Korea | Breeding line | *Indica* |
| Acc-006 | CT9993-5-10-1-M | Colombia | Introduction | *Indica* |
| Acc-007 | RATHAL | Sri lanka | Introduction | *admixture* |
| Acc-008 | TCHAMPA | Iran | Introduction | *Indica* |
| Acc-009 | Jejubukjeju-2002-99 | Korea | Weedy | *japonica* |
| Acc-010 | Jejubukjeju-2002-171 | Korea | Weedy | *Indica* |
| Acc-011 | Jejubukjeju-2002-340 | Korea | Weedy | *japonica* |
| Acc-012 | Jejubukjeju-2002-420 | Korea | Weedy | *japonica* |
| Acc-013 | Jejubukjeju-2002-521 | Korea | Weedy | *Indica* |
| Acc-014 | Incheonkanghwasujip-16 | Korea | Weedy | *japonica* |
| Acc-015 | Muando | Korea | Landrace | *japonica* |
| Acc-016 | Dadajo | Korea | Landrace | *japonica* |
| Acc-017 | OKCHEONG | Korea | Landrace | *japonica* |
| Acc-018 | Sando | Korea | Landrace | *japonica* |
| Acc-019 | Batnarak | Korea | Landrace | *japonica* |
| Acc-020 | Orido | Korea | Landrace | *japonica* |
| Acc-021 | Saducho | Korea | Landrace | *Indica* |
| Acc-022 | SEON | Korea | Landrace | *Indica* |
| Acc-023 | Hanyangjo | Korea | Landrace | *Indica* |
| Acc-024 | Inbujido | Korea | Landrace | *japonica* |
| Acc-025 | Beobpanhwa | Korea | Landrace | *japonica* |
| Acc-026 | JANMOCHAL | Korea | Landrace | *japonica* |
| Acc-027 | Pyodo | Korea | Landrace | *japonica* |
| Acc-028 | YULJOJO | Korea | Landrace | *japonica* |
| Acc-029 | Samgyeongjo | Korea | Landrace | *japonica* |
| Acc-030 | BAEKGOGNA | Korea | Landrace | *Indica* |
| Acc-031 | MONDONCHALBYEO(Mongdonjaerae) | Korea | Landrace | *admixture* |
| Acc-032 | AGBEDE | Nigeria | Introduction | *japonica* |
| Acc-033 | ANBAW C7 | Brunei | Introduction | *japonica* |
| Acc-034 | BALA | India | Introduction | *Indica* |
| Acc-035 | BELLARDONE | Egypt | Introduction | *japonica* |
| Acc-036 | CHIEM CHANK | Vietnam | Introduction | *Indica* |
| Acc-037 | DHARIAL | Surinam | Introduction | *Indica* |
| Acc-038 | DULAR | India | Introduction | *Indica* |
| Acc-039 | IR38 | Philippines | Introduction | *Indica* |
| Acc-040 | MAGNOLIA | USA | Introduction | *japonica* |
| Acc-041 | MALA | Bangladesh | Introduction | *Indica* |
| Acc-042 | Mushkan 41 | Philippines | Introduction | *admixture* |
| Acc-043 | PUKHI | Pakistan | Introduction | *Indica* |
| Acc-044 | Red Rice | Iran | Introduction | *Indica* |
| Acc-045 | TUN SART | Vietnam | Introduction | *japonica* |
| Acc-046 | VICTORIA F.A | Afghanistan | Introduction | *japonica* |
| Acc-047 | Bai Cyue Hwa Lue | Taiwan | Introduction | *Indica* |
| Acc-048 | Yangmyeon | Taiwan | Introduction | *Indica* |
| Acc-049 | BELLE PATNA | USA | Introduction | *Indica* |
| Acc-050 | Pyeongbuk 3 | North Korea | Introduction | *japonica* |
| Acc-051 | YUPUL | Liberia | Introduction | *japonica* |
| Acc-052 | Liman Belozernij | Russia | Introduction | *japonica* |
| Acc-053 | KAGI | India | Introduction | *Indica* |
| Acc-054 | HATADANI | Sri lanka | Introduction | *Indica* |
| Acc-055 | TAI MOCHITO | Thailand | Introduction | *japonica* |
| Acc-056 | WAIKYAKUSHI | Taiwan | Introduction | *Indica* |
| Acc-057 | UPLAND | Nigeria | Introduction | *japonica* |
| Acc-058 | NEWREX | USA | Introduction | *japonica* |
| Acc-059 | Doan Guang Hwa Lue | Taiwan | Introduction | *japonica* |
| Acc-060 | Wu Cyue | Taiwan | Introduction | *Indica* |
| Acc-061 | BINATO | Philippines | Introduction | *Indica* |
| Acc-062 | Avangard | Uzbekistan | Introduction | *japonica* |
| Acc-063 | Debzera | Uzbekistan | Introduction | *japonica* |
| Acc-064 | HAWM SUPAN | Thailand | Introduction | *Indica* |
| Acc-065 | Ssal Byeo 22 | Korea | Weedy | *japonica* |
| Acc-066 | Urasan | Japan | Introduction | *japonica* |
| Acc-067 | XI GUA BAI | China | Introduction | *Indica* |
| Acc-068 | YUNG YUEN CHUEN ZIM | China | Introduction | *Indica* |
| Acc-069 | KENG CHI JU | China | Introduction | *japonica* |
| Acc-070 | QUA 77 WUAN-DAU | China | Introduction | *Indica* |
| Acc-071 | CHIH-TSAO-HE | China | Introduction | *Indica* |
| Acc-072 | HSIANG-HA-TSAN | China | Introduction | *Indica* |
| Acc-073 | Cheongdo-donggok-4 | Korea | Weedy | *Indica* |
| Acc-074 | Golyeong-2 | Korea | Weedy | *japonica* |
| Acc-075 | Golyeong-6 | Korea | Weedy | *japonica* |
| Acc-076 | Danyang-7 | Korea | Weedy | *japonica* |
| Acc-077 | Danyang-38 | Korea | Weedy | *japonica* |
| Acc-078 | Hwaseong-5 | Korea | Weedy | *japonica* |
| Acc-079 | Gou 405 | JPN | Introduction | *japonica* |
| Acc-080 | Baeksami | CHN | Introduction | *japonica* |
| Acc-081 | AKAINE | JPN | Introduction | *japonica* |
| Acc-082 | Syarebyeo-61-1-B | Korea | Weedy | *japonica* |
| Acc-083 | Syalebyeo-94-1-B | Korea | Weedy | *japonica* |
| Acc-084 | Syalebyeo-163-1-B | Korea | Weedy | *japonica* |
| Acc-085 | Milyang 50 | Korea | Breeding line | *Indica* |
| Acc-086 | Yeongpung Byeo | Korea | Breeding line | *Indica* |
| Acc-087 | Iri 336 | Korea | Breeding line | *japonica* |
| Acc-088 | Mihyang Byeo | Korea | Breeding line | *japonica* |
| Acc-089 | MOROBEREKAN | Nepal | Introduction | *japonica* |
| Acc-090 | Jejubukjeju-2002-115 | Korea | Weedy | *japonica* |
| Acc-091 | Jejubukjeju-2002-550 | Korea | Weedy | *japonica* |
| Acc-092 | Jejubukjeju-2002-561 | Korea | Weedy | *japonica* |
| Acc-093 | Huindadak | Korea | Landrace | *japonica* |
| Acc-094 | Jotajo | Korea | Landrace | *japonica* |
| Acc-095 | Pocheon Jangmang Mebyeo | Korea | Landrace | *japonica* |
| Acc-096 | Dongo Byeo | Korea | Landrace | *japonica* |
| Acc-097 | Seorianjeunbaengi | Korea | Landrace | *japonica* |
| Acc-098 | Neul Byeo | Korea | Landrace | *japonica* |
| Acc-099 | Jwiippari Byeo | Korea | Landrace | *japonica* |
| Acc-100 | Jeongjonghwa | Korea | Landrace | *japonica* |
| Acc-101 | Sodujo | Korea | Landrace | *japonica* |
| Acc-102 | Sando | Korea | Landrace | *japonica* |
| Acc-103 | Bori Byeo | Korea | Landrace | *japonica* |
| Acc-104 | Naengjo | Korea | Landrace | *japonica* |
| Acc-105 | BIKOM | Nigeria | Introduction | *admixture* |
| Acc-106 | British Honduras Creole | Honduras | Introduction | *japonica* |
| Acc-107 | CARTUNA | USA | Introduction | *japonica* |
| Acc-108 | SAHAK | Puerto rico | Introduction | *Indica* |
| Acc-109 | TAICHUNG-WOO-TSAN | Taiwan | Introduction | *Indica* |
| Acc-110 | ZILANICA | Surinam | Introduction | *admixture* |
| Acc-111 | Xao Bai Mang Sue Dao | Taiwan | Introduction | *japonica* |
| Acc-112 | Di jiao wu jian | Taiwan | Introduction | *Indica* |
| Acc-113 | SPIN MERE | Afghanistan | Introduction | *Indica* |
| Acc-114 | Norin 22 | Japan | Introduction | *japonica* |
| Acc-115 | Ssalbyeo 16 | Korea | Weedy | *japonica* |
| Acc-116 | NIAN CHI SHI | China | Introduction | *Indica* |
| Acc-117 | SUNG PAN TAO | China | Introduction | *japonica* |
| Acc-118 | TSONG-GAN-SHUN | China | Introduction | *Indica* |
| Acc-119 | SAN-LI-SHUN | China | Introduction | *Indica* |
| Acc-120 | YANG-SHA-TSAN | China | Introduction | *Indica* |
| Acc-121 | Chungdo 23 | Korea | Weedy | *Indica* |
| Acc-122 | Chungdo Hwayang 12 | Korea | Weedy | *Indica* |
| Acc-123 | Chungdo Hwayang 14 | Korea | Weedy | *Indica* |
| Acc-124 | Sungju 3 | Korea | Weedy | *Indica* |
| Acc-125 | Jangsung 1 | Korea | Weedy | *Indica* |
| Acc-126 | Soonchun 5 | Korea | Weedy | *Indica* |
| Acc-127 | Daegu Damti 6-2 | Korea | Weedy | *japonica* |
| Acc-128 | Guechang 15 | Korea | Weedy | *japonica* |
| Acc-129 | Danyang 9 | Korea | Weedy | *japonica* |
| Acc-130 | Chungsongaengmi 4 | Korea | Weedy | *japonica* |
| Acc-131 | Suhyeonghando | China | Introduction | *Indica* |
| Acc-132 | Xiao zao huang | China | Introduction | *japonica* |
| Acc-133 | Namseon 34 | Korea | Breeding line | *japonica* |
| Acc-134 | Namseon 126 | Korea | Breeding line | *japonica* |
| Acc-135 | Milyang 88 | Korea | Breeding line | *japonica* |
| Acc-136 | Dudo | Korea | Landrace | *japonica* |
| Acc-137 | IR40 | Philippines | Introduction | *Indica* |
| Acc-138 | Jinbu Byeo | Korea | Breeding line | *japonica* |
| Acc-139 | Palgong Byeo | Korea | Breeding line | *japonica* |
| Acc-140 | Hopyung | Korea | Breeding line | *japonica* |
| Acc-141 | Dongjin Chal Byeo | Korea | Breeding line | *japonica* |
| Acc-142 | Gopum | Korea | Breeding line | *japonica* |
| Acc-143 | Unkwang | Korea | Breeding line | *japonica* |
| Acc-144 | Haiami | Korea | Breeding line | *japonica* |
| Acc-145 | Samgwang | Korea | Breeding line | *japonica* |
| Acc-146 | Hopum | Korea | Breeding line | *japonica* |
| Acc-147 | Chilbo | Korea | Breeding line | *japonica* |
| Acc-148 | Ilpum Byeo | Korea | Breeding line | *japonica* |
| Acc-149 | Chucheong Byeo | Japan | Introduction | *japonica* |
| Acc-150 | Junam Byeo | Korea | Breeding line | *japonica* |
| Acc-151 | Shindongjin Byeo | Korea | Breeding line | *japonica* |
| Acc-152 | Odae Byeo | Korea | Breeding line | *japonica* |
| Acc-153 | Nam Il | Korea | Breeding line | *japonica* |
| Acc-154 | Hwaseong Byeo | Korea | Breeding line | *japonica* |
| Acc-155 | Boramchan | Korea | Breeding line | *japonica* |
| Acc-156 | Hwayeong Byeo | Korea | Breeding line | *japonica* |
| Acc-157 | Dongjin Byeo | Korea | Breeding line | *japonica* |
| Acc-158 | Nakdong Byeo | Korea | Breeding line | *japonica* |
| Acc-159 | Giho Byeo | Korea | Breeding line | *japonica* |
| Acc-160 | Hwacheong | Korea | Breeding line | *japonica* |
| Acc-161 | Jopyeong | Korea | Breeding line | *japonica* |
| Acc-162 | Chinnong | Korea | Breeding line | *japonica* |
| Acc-163 | Hanareum | Korea | Breeding line | *Indica* |
| Acc-164 | Dasan Byeo | Korea | Breeding line | *Indica* |
| Acc-165 | Milyang 23 | Korea | Breeding line | *Indica* |
| Acc-166 | Samgang Byeo | Korea | Breeding line | *Indica* |
| Acc-167 | Nokyang | Korea | Breeding line | *japonica* |
| Acc-168 | Tongil | Korea | Breeding line | *Indica* |
| Acc-169 | Unbong Byeo | Korea | Breeding line | *japonica* |
| Acc-170 | Sinunbong Byeo | Korea | Breeding line | *japonica* |
| Acc-171 | Sambaek Byeo | Korea | Breeding line | *japonica* |
| Acc-172 | Junghwa Byeo | Korea | Breeding line | *japonica* |
| Acc-173 | Geuru Byeo | Korea | Breeding line | *japonica* |
| Acc-174 | Inwol Byeo | Korea | Breeding line | *japonica* |
| Acc-175 | Sangmibyeo | Korea | Breeding line | *japonica* |
| Acc-176 | Geumo Byeo | Korea | Breeding line | *japonica* |
| Acc-177 | Jinbuol Byeo | Korea | Breeding line | *japonica* |
| Acc-178 | Jinmi Byeo | Korea | Breeding line | *japonica* |
| Acc-179 | Heugjinju Byeo | Korea | Breeding line | *japonica* |
| Acc-180 | Jeogjinju Byeo | Korea | Breeding line | *japonica* |
| Acc-181 | Naepung Byeo | Korea | Breeding line | *japonica* |
| Acc-182 | Donghae Byeo | Korea | Breeding line | *japonica* |
| Acc-183 | Seoan Byeo | Korea | Breeding line | *japonica* |
| Acc-184 | Gancheok Byeo | Korea | Breeding line | *japonica* |
| Acc-185 | Juan Byeo | Korea | Breeding line | *japonica* |
| Acc-186 | Yeonghae Byeo | Korea | Breeding line | *japonica* |
| Acc-187 | Sobi Byeo | Korea | Breeding line | *japonica* |
| Acc-188 | Haepyeong Byeo | Korea | Breeding line | *japonica* |
| Acc-189 | Sinseonchal Byeo | Korea | Breeding line | *japonica* |
| Acc-190 | Daelip Byeo 1 | Korea | Breeding line | *japonica* |
| Acc-191 | Seolhyangchal Byeo | Korea | Breeding line | *japonica* |
| Acc-192 | Ilmi Byeo | Korea | Breeding line | *japonica* |
| Acc-193 | Nampyeong Byeo | Korea | Breeding line | *japonica* |
| Acc-194 | Gyehwa Byeo | Korea | Breeding line | *japonica* |
| Acc-195 | Yeongnam Byeo | Korea | Breeding line | *japonica* |
| Acc-196 | Yangjo Byeo | Korea | Breeding line | *japonica* |
| Acc-197 | Aranghangchal Byeo | Korea | Breeding line | *japonica* |
| Acc-198 | Heugnam Byeo | Korea | Breeding line | *japonica* |
| Acc-199 | Goamy Byeo | Korea | Breeding line | *japonica* |
| Acc-200 | Manmi | Korea | Breeding line | *japonica* |
| Acc-201 | Heugkwang Byeo | Korea | Breeding line | *japonica* |
| Acc-202 | Norinmochi 1 | Korea | Breeding line | *japonica* |
| Acc-203 | Sangnambat Byeo | Korea | Breeding line | *japonica* |
| Acc-204 | Anda Byeo | Korea | Breeding line | *Indica* |
| Acc-205 | Taebaeg Byeo | Korea | Breeding line | *Indica* |
| Acc-206 | Gaya Byeo | Korea | Breeding line | *Indica* |
| Acc-207 | Baegyang Byeo | Korea | Breeding line | *Indica* |
| Acc-208 | Cheong Cheong Byeo | Korea | Breeding line | *Indica* |
| Acc-209 | Jungwon Byeo | Korea | Breeding line | *Indica* |
| Acc-210 | Nampung Byeo | Korea | Breeding line | *Indica* |
| Acc-211 | Hangangchal | Korea | Breeding line | *Indica* |
| Acc-212 | Goun | Korea | Breeding line | *japonica* |
| Acc-213 | Pungmi | Korea | Breeding line | *japonica* |
| Acc-214 | Boseogchal | Korea | Breeding line | *japonica* |
| Acc-215 | Hanmaeum | Korea | Breeding line | *japonica* |
| Acc-216 | Hwasin 1 | Korea | Breeding line | *japonica* |
| Acc-217 | Onnuri | Korea | Breeding line | *japonica* |
| Acc-218 | Gangbaek | Korea | Breeding line | *japonica* |
| Acc-219 | Hwangkeumnuri | Korea | Breeding line | *japonica* |
| Acc-220 | Dami | Korea | Breeding line | *japonica* |
| Acc-221 | Hongjinju | Korea | Breeding line | *japonica* |
| Acc-222 | Sinmyungheugchal | Korea | Breeding line | *japonica* |
| Acc-223 | Hoban | Korea | Breeding line | *japonica* |
| Acc-224 | Heugseol | Korea | Breeding line | *japonica* |
| Acc-225 | Danmi | Korea | Breeding line | *japonica* |
| Acc-226 | Nokwonchal | Korea | Breeding line | *japonica* |
| Acc-227 | Younghojinmi | Korea | Breeding line | *japonica* |
| Acc-228 | Honong | Korea | Breeding line | *japonica* |
| Acc-229 | Joun | Korea | Breeding line | *japonica* |
| Acc-230 | Wolbaek | Korea | Breeding line | *japonica* |
| Acc-231 | Gangchan | Korea | Breeding line | *japonica* |
| Acc-232 | Shinbaeg | Korea | Breeding line | *japonica* |
| Acc-233 | Geongganghongmi | Korea | Breeding line | *japonica* |
| Acc-234 | Sodami | Korea | Breeding line | *japonica* |
| Acc-235 | Sukwang | Korea | Breeding line | *japonica* |
| Acc-236 | Seonhyangheukmi | Korea | Breeding line | *japonica* |
| Acc-237 | Pungok | Korea | Breeding line | *japonica* |
| Acc-238 | Wase Gingbouzu | Korea | Breeding line | *Indica* |
| Acc-239 | Palgoeng | Korea | Breeding line | *japonica* |
| Acc-240 | Jinheung | Korea | Breeding line | *japonica* |
| Acc-241 | Milseong | Korea | Breeding line | *japonica* |
| Acc-242 | Yusin | Korea | Breeding line | *Indica* |
| Acc-243 | Satbyeol Byeo | Korea | Breeding line | *Indica* |
| Acc-244 | Milyang 42 | Korea | Breeding line | *Indica* |
| Acc-245 | Dobong | Korea | Breeding line | *japonica* |
| Acc-246 | Seolag Byeo | Korea | Breeding line | *japonica* |
| Acc-247 | Samnam Byeo | Korea | Breeding line | *japonica* |
| Acc-248 | Seomjin Byeo | Korea | Breeding line | *japonica* |
| Acc-249 | Youngdeok | Korea | Breeding line | *japonica* |
| Acc-250 | Seohae | Korea | Breeding line | *japonica* |
| Acc-251 | Mimyeon | Korea | Breeding line | *Indica* |
| Acc-252 | MS11 | Korea | Breeding line | *japonica* |
| Acc-253 | KOSHIHIKARI | Japan | Introduction | *japonica* |
| Acc-254 | Sobaeg Byeo | Korea | Breeding line | *japonica* |
| Acc-255 | Sangju Byeo | Korea | Breeding line | *japonica* |
| Acc-256 | Samcheon Byeo | Korea | Breeding line | *japonica* |
| Acc-257 | Munjang Byeo | Korea | Breeding line | *japonica* |
| Acc-258 | Taebong Byeo | Korea | Breeding line | *japonica* |
| Acc-259 | Dunnae Byeo | Korea | Breeding line | *japonica* |
| Acc-260 | Saesangju | Korea | Breeding line | *japonica* |
| Acc-261 | Manchu Byeo | Korea | Breeding line | *japonica* |
| Acc-262 | Nongan Byeo | Korea | Breeding line | *japonica* |
| Acc-263 | Sura Byeo | Korea | Breeding line | *japonica* |
| Acc-264 | Bonggwang | Japan | Introduction | *japonica* |
| Acc-265 | Hwaseonchal Byeo | Korea | Breeding line | *japonica* |
| Acc-266 | Dongan Byeo | Korea | Breeding line | *japonica* |
| Acc-267 | Daesan Byeo | Korea | Breeding line | *japonica* |
| Acc-268 | Nongho Byeo | Korea | Breeding line | *japonica* |
| Acc-269 | Manguem Byeo | Korea | Breeding line | *japonica* |
| Acc-270 | Saegyehwa | Korea | Breeding line | *japonica* |
| Acc-271 | Manweol Byeo | Korea | Breeding line | *japonica* |
| Acc-272 | Hyangmi Byeo 1 | Korea | Breeding line | *Indica* |
| Acc-273 | Hwangkeumbora | Korea | Breeding line | *japonica* |
| Acc-274 | Cheonga | Korea | Breeding line | *japonica* |
| Acc-275 | Cheongdam | Korea | Breeding line | *japonica* |
| Acc-276 | Keunseom | Korea | Breeding line | *Indica* |
| Acc-277 | Saenuri | Korea | Breeding line | *japonica* |
| Acc-278 | Hwanggeumnodeul | Korea | Breeding line | *japonica* |
| Acc-279 | Cheongan | Korea | Breeding line | *japonica* |
| Acc-280 | Deuraechan | Korea | Breeding line | *japonica* |
| Acc-281 | Jinbaek | Korea | Breeding line | *japonica* |
| Acc-282 | Cheongnam | Korea | Breeding line | *japonica* |
| Acc-283 | Suan | Korea | Breeding line | *japonica* |
| Acc-284 | Dongbo | Korea | Breeding line | *japonica* |
| Acc-285 | Seolemi | Korea | Breeding line | *japonica* |
| Acc-286 | Jungsaenggold | Korea | Breeding line | *japonica* |
| Acc-287 | Saeilmi | Korea | Breeding line | *japonica* |
| Acc-288 | Seokwang | Korea | Breeding line | *japonica* |
| Acc-289 | Saenara | Korea | Breeding line | *japonica* |
| Acc-290 | Paldal | Korea | Breeding line | *japonica* |
| Acc-291 | Nongbaeg | Korea | Breeding line | *japonica* |
| Acc-292 | Chupung Byeo | Korea | Breeding line | *Indica* |
| Acc-293 | Kwanak Byeo | Korea | Breeding line | *japonica* |
| Acc-294 | Seonam Byeo | Korea | Breeding line | *japonica* |
| Acc-295 | Daw Dam | Korea | Breeding line | *japonica* |

^a^ The type was determined by PowerCore (Kim et al. 2007). ^b^ Defined by ADMIXTURE (Alexander et al. 2009) when K = 2 using nuclear genome SNP data.
