# Supplementary material for: Rice Chloroplast Genome Variation Architecture and Phylogenetic Dissection in Diverse Oryza Species Assessed by Whole-Genome Resequencing
Source: Rice (N Y). 2016 Oct 18;9:57. doi: 10.1186/s12284-016-0129-y (PMC5069220; doi:10.1186/s12284-016-0129-y)
Supplement: Additional file 8: Table S6. — Fifty cultivated and wild rice accessions and 38 African rice (including 19 African cultivated rice and 19 African wild rice) accessions used in the chloroplast genome study. (DOCX 21 kb) [file 12284_2016_129_MOESM8_ESM.docx]

**Table S6.** Fifty cultivated and wild rice accessions and 19 accessions of African rice used for current chloroplast genome study.

| **No.** | **Accession name** | **Accession ID *** | **Status** | **Origin** | **Variety group**** |
| --- | --- | --- | --- | --- | --- |
| 50 cultivated and wild rice | | |  |  |  |
| 1 | Mehr | IRGC 12883 | Landrace | Iran | AUS |
| 2 | Kalamkati | IRGC 45975 | Landrace | India | AUS |
| 3 | Jhona 349 | IRGC 6307 | Landrace | India | AUS |
| 4 | DZ78 | IRGC 8555 | Landrace | Bangladesh | AUS |
| 5 | Binulawan | IRGC 26872 | Landrace | Philippines | TRJ |
| 6 | Leung Pratew | IRGC 27762 | Landrace | Thailand | IND |
| 7 | IR 36 | IRGC 30416 | Improve | Brazil | IND |
| 8 | Popot 165 | IRGC 43545 | Landrace | Indonesia | IND |
| 9 | Ai-Chiao-Hong | IRGC 51250 | Landrace | China | IND |
| 10 | Guan-Yin-Tsan | IRGC 51300 | Landrace | China | IND |
| 11 | Gie 57 | IRGC 8231 | Landrace | Vietnam | IND |
| 12 | TD2 | IRGC 9148 | Elite | Thailand | IND |
| 13 | JC91 | IRGC 9177 | Elite | India | IND |
| 14 | Ta Hung Ku | IRGC 1107 | Landrace | China | TEJ |
| 15 | Haginomae Mochi | IRGC 2540 | Elite | Japan | TEJ |
| 16 | Darmali | IRGC 27630 | Landrace | Nepal | TEJ |
| 17 | Phudugey | IRGC 32399 | Landrace | Bhutan | TEJ |
| 18 | Norin 20 | IRGC 418 | Landrace | Japan | TEJ |
| 19 | Chodongji | IRGC 55471 | Landrace | South Korea | TEJ |
| 20 | Mansaku | IRGC 8191 | Landrace | Japan | TEJ |
| 21 | Nipponbare | NP | Elite | Japan | TEJ |
| 22 | Maintmolotsy | IRGC 11010 | Elite | Madagascar | TRJ |
| 23 | Jambu | IRGC 17757 | Landrace | Indonesia | TRJ |
| 24 | Miriti | IRGC 25901 | Landrace | Bangladesh | IND |
| 25 | AZUCENA | IRGC 328 | Landrace | Philippines | TRJ |
| 26 | NPE 844 | IRGC 38698 | Landrace | Pakistan | TRJ |
| 27 | Arias | IRGC 43325 | Landrace | Indonesia | TRJ |
| 28 | Gotak Gatik | IRGC 43397 | Landrace | Indonesia | TRJ |
| 29 | Trembese | IRGC 43675 | Landrace | Indonesia | TRJ |
| 30 | Canella De Ferro | IRGC 50448 | Elite | Brazil | TRJ |
| 31 | Lemont | IRGC 66756 | Elite | TX,USA | TRJ |
| 32 | Davao | IRGC 8244 | Landrace | Philippines | TRJ |
| 33 | Kitrana 508 | IRGC 12793 | Elite | Madagascar | ARO |
| 34 | Bico Branco | IRGC 38994 | Elite | Brazil | ARO |
| 35 | JC101 | IRGC 9060 | Elite | India | ARO |
| 36 | JC111 | IRGC 9062 | Elite | India | ARO |
| 37 | Firooz | RA 4952 | Landrace | Iran | ARO |
| 38 | KUI SALI | IRGC 31856 | Landrace | Bangladesh | ARO |
| 39 | HAISHA CAMAN | IRGC 60542 | Landrace | Bangladesh | IV |
| 40 | BADAL 89 | IRGC 6513 | Landrace | Bangladesh | III |
| 41 | 042/87/34 | IRGC 105327 | wild | India | *nivara* |
| 42 | MV 89-80 | IRGC 106105 | wild | India | *nivara* |
| 43 | L 89-12 | IRGC 106154 | wild | Vientiane, Laos | *nivara* |
| 44 | HK 47 | IRGC 80470 | wild | India | *nivara* |
| 45 | CA 97-053 | IRGC 89215 | wild | Cambodia | *nivara* |
| 46 | PADI PADIAN | IRGC 105958 | wild | Indonesia | *rufipogon* |
| 47 | DAL DHAN | IRGC 105960 | wild | Bangladesh | *rufipogon* |
| 48 | VOC4 | VOC4 | wild | Nepal | *rufipogon* |
| 49 | P46 | P46 | wild | China | *rufipogon* |
| 50 | Yuan 3-9 | Yuan 3-9 | wild | China | *rufipogon* |
| *O. glaberrima* accessions | |  |  |  |  |
| 1 | - | IRGC103469 | cultivar | Burkina Faso | *O. glaberrima* |
| 2 | - | TOG5923 | cultivar | Liberia | *O. glaberrima* |
| 3 | - | TOG5949 | cultivar | Liberia | *O. glaberrima* |
| 4 | - | TOG5467 | cultivar | Nigeria | *O. glaberrima* |
| 5 | - | TOG5457 | cultivar | Nigeria | *O. glaberrima* |
| 6 | - | TOG7025 | cultivar | Sierra Leone | *O. glaberrima* |
| 7 | - | TOG7102 | cultivar | Mali | *O. glaberrima* |
| 8 | - | IRGC*67563* | cultivar | Ghana | *O. glaberrima* |
| 9 | - | IRGC68939 | cultivar | Madagascar | *O. glaberrima* |
| 10 | - | IRGC68976 | cultivar | Guyana | *O. glaberrima* |
| 11 | - | IRGC75500 | cultivar | Burkina Faso | *O. glaberrima* |
| 12 | - | IRGC96841 | cultivar | Zimbabwe | *O. glaberrima* |
| 13 | - | IRGC101049 | cultivar | Senegal | *O. glaberrima* |
| 14 | - | IRGC103472 | cultivar | Burkina Faso | *O. glaberrima* |
| 15 | - | IRGC103520 | cultivar | Mali | *O. glaberrima* |
| 16 | - | IRGC103632 | cultivar | Mali | *O. glaberrima* |
| 17 | - | IRGC103937 | cultivar | Liberia | *O. glaberrima* |
| 18 | - | IRGC104206 | cultivar | Ghana | *O. glaberrima* |
| 19 | - | IRGC104574 | cultivar | Mali | *O. glaberrima* |
| *O. barthii* accessions | |  |  |  |  |
| 1 | - | IRGC100122 | wild | Gambia | *O. barthii* |
| 2 | - | IRGC100931 | wild | Mali | *O. barthii* |
| 3 | - | IRGC100934 | wild | Mali | *O. barthii* |
| 4 | - | IRGC103895 | wild | Senegal | *O. barthii* |
| 5 | - | IRGC104084 | wild | Nigeria | *O. barthii* |
| 6 | - | IRGC104119 | wild | Chad | *O. barthii* |
| 7 | - | IRGC105608 | wild | Cameroon | *O. barthii* |
| 8 | - | IRGC106234 | wild | Sierra Leone | *O. barthii* |
| 9 | - | IRGC103912 | wild | Tanzania | *O. barthii* |
| 10 | - | WAB0028952 | wild | Zambia | *O. barthii* |
| 11 | - | WAB0028903 | wild | Zambia | *O. barthii* |
| 12 | - | WAB0028980 | wild | Mali | *O. barthii* |
| 13 | - | WAB0028987 | wild | Nigeria | *O. barthii* |
| 14 | - | WAB0028958 | wild | Mali | *O. barthii* |
| 15 | - | WAB0028976 | wild | Mali | *O. barthii* |
| 16 | - | WAB0028979 | wild | Mali | *O. barthii* |
| 17 | - | WAB0028992 | wild | Chad | *O. barthii* |
| 18 | - | WAB0028938 | wild | Nigeria | *O. barthii* |
| 19 | - | WAB0030151 | wild | Chad | *O. barthii* |

* Theser accessions were obtained from the Genetic Resources Center of the International Rice Research Institute (IRRI) at Los Banos, Philippines or collected by the authors (Xu et al, 2012). Accession numbers starting with “TOG” and “WAB” were obtained from African Rice Center (Wang et al, 2014). The remaining accessions were obtained from the International Rice Research Institute (Xu et al, 2012, Wang et al, 2014).

** On the basis of Garris et al. (2005), five distinct variety groups were recognized for cultivated rice: aus (AUS), indica (IND), aromatic (ARO), temperate japonica
